# Effects of human intravenous immunoglobulin on amyloid pathology and neuroinflammation in a mouse model of Alzheimer’s disease

**DOI:** 10.1186/1742-2094-9-105

**Published:** 2012-05-29

**Authors:** Lakshman Puli, Yuriy Pomeshchik, Katja Olas, Tarja Malm, Jari Koistinaho, Heikki Tanila

**Affiliations:** 1 A. I. Virtanen Institute, University of Eastern Finland, Kuopio, Finland; 2 Department of Clinical Medicine/Oncology, Kuopio University Hospital, Kuopio, Finland; 3 Department of Clinical Medicine/Neurology, Kuopio University Hospital, Kuopio, Finland; 4 Baxter Innovations GmbH, Vienna, Austria

**Keywords:** Human intravenous immunoglobulin, Amyloid pathology, Microglia, Neuroinflammation, Alzheimer’s disease

## Abstract

****Background**:**

Human intravenous immunoglobulin (hIVIG) preparation is indicated for treating primary immunodeficiency disorders associated with impaired humoral immunity. hIVIG is known for its anti-inflammatory properties and a decent safety profile. Therefore, by virtue of its constituent natural anti-amyloid beta antibodies and anti-inflammatory effects, hIVIG is deemed to mediate beneficial effects to patients of Alzheimer’s disease (AD). Here, we set out to explore the effects of hIVIG in a mouse model of AD.

****Methods**:**

We treated APP/PS1dE9 transgenic and wild-type mice with weekly injections of a high hIVIG dose (1 g/kg) or saline for 3 or 8 months. Treatment effect on brain amyloid pathology and microglial reactivity was assessed by ELISA, immunohistochemistry, RT-PCR, and confocal microscopy.

****Results**:**

We found no evidence for reduction in Aβ pathology; instead 8 months of hIVIG treatment significantly increased soluble levels of Aβ40 and Aβ42. In addition, we noticed a significant reduction in CD45 and elevation of Iba-1 markers in specific sub-populations of microglial cells. Long-term hIVIG treatment also resulted in significant suppression of TNF-α and increase in doublecortin positive adult-born neurons in the dentate gyrus.

****Conclusions**:**

Our data indicate limited ability of hIVIG to impact amyloid burden but shows changes in microglia, pro-inflammatory gene expression, and neurogenic effects. Immunomodulation by hIVIG may account for its beneficial effect in AD patients.

## **Background**

Alzheimer’s disease (AD) is a progressive neurodegenerative disease that poses enormous social, economical, and emotional burden on the resources of both developed and developing countries. Presence of amyloid-β (Aβ) deposits, neurofibrillary tangles, and neuronal loss in brain areas responsible for maintenance of cognitive functions are primary hallmarks of AD. According to amyloid cascade hypothesis [1], Aβ protein inside brain unleashes a cascade of downstream events that ultimately results in loss of synapses and neurons. However, it is still not clear whether Aβ is a trigger or a driver of AD pathology. One important consequence of Aβ deposition in the brain tissue is neuroinflammation. Phagocytic or macropinocytic activation of resident microglia and macrophages around amyloid deposits can be seen as a protective response, since there is substantial evidence for their involvement in the clearance of extracellular Aβ [2]. On the other hand, neuroinflammation may as well facilitate development of amyloid plaque pathology [3]. In addition, release of pro-inflammatory cytokines may contribute to the dysfunction and neurodegeneration. For instance, pro-inflammatory cytokines TNF-α and IL-1β impair synaptic plasticity and thereby can induce memory impairment, while anti-inflammatory cytokine IL-4 has an opposite effect [4,5]. There is evidence that these two effects of microglial activation may be differentially regulated. For instance, while phagocytosis of Aβ is the dominant response in young mice with amyloid deposits, in aged mice phagocytosis associated gene expression decreases while that of pro-inflammatory cytokines increases [6]. There is also evidence that despite accumulation around amyloid plaques microglia seem to be incapable of Aβ phagocytosis unless specifically activated [7]. One way to induce putative beneficial activation in microglia is either by active or passive immunotherapy. Active immunization with Aβ has proved to be an efficient way to reduce brain amyloid load in APP transgenic mouse models of AD [8-10], but their first clinical trials needed to be discontinued due to development of fulminant meningo-encephalitis in a considerable number of patients [11]. Also passive immunization with monoclonal antibodies reactive to N-terminal, middle portion, or C-terminal of Aβ protein have proven to be effective in AD mouse models [12-14] and are currently in various phases of clinical development. However, also passive immunotherapy bears the risk of microhemorrhages [15]. In addition, all clinical trials so far with monoclonal Aβ antibodies have yielded meager benefit for the patients [16].

Human intravenous immunoglogulins (hIVIG), a spectrum of polyclonal natural antibodies, have a long history of being a safe and effective treatment for certain neurological conditions such as Guillain-Barré syndrome [17]. Constituent antibodies of hIVIG are reactive to a plethora of inflammatory proteins and their mediators. In addition, a small fraction of antibodies in hIVIG are reactive to Aβ protein [18], which has encouraged clinical trials to test hIVIG in AD patients. Indeed, several studies with a small patient number have produced promising effects on Aβ levels in the CSF and also some positive effects on cognitive status of AD patients [19,20]. Mechanistically, hIVIG is hypothesized to promote Aβ clearance by virtue of its constituent anti-Aβ antibodies, which can account for its beneficial effect in AD patients. In addition, due to its anti-inflammatory properties hIVIG may modulate the neuroinflammatory reaction around amyloid plaques towards neuroprotective direction and thereby mediate beneficial effects in AD patients. Clearly, there is an urgent need to test the mechanisms of hIVIG action in an animal model of AD.

Our previous studies [21] indicate that it is indeed possible to study the biological effects of hIVIG in the human CNS using a mouse model. As expected, mice develop neutralizing antibodies towards human IgG, but prolonged treatments produced immunotolerance. Furthermore, hIVIG is capable to cross the mouse blood–brain barrier and reach significant concentrations especially in the septal (dorsal) hippocampus aligning the lateral ventricles. We also reported that systemic administration results in decoration of amyloid plaques with hIVIG in the hippocampus [21]. In the current study, we treated 4-month-old APP/PS1dE9 mice with weekly injections of a high hIVIG dose (1 g/kg) for either 3 months or 8 months and assessed its impact on amyloid pathology and neuroinflammation. These mice develop amyloid plaques with a dense Congo red positive core and diffuse outer layer, closely resembling amyloid plaques in AD brains [22]. The first plaques appear in the neocortex and hippocampus around 4 months of age, followed by a rapid accumulation until 11 to 12 months of age [23]. The longer treatment thus covered the entire period of rapid amyloid plaque formation. Nevertheless, even the longer hIVIG treatment did not reduce amyloid deposition but induced selective immunomodulation and enhanced neurogenesis, which both may be significant for the beneficial clinical effects of hIVIG in AD patients.

## **Materials and Methods**

### **Animals and experimental design**

Four-month-old female APPswe/PS1dE9 (APP/PS1dE9) transgenic and their wild-type littermates were used. APP/PS1dE9 mice harbor human APPswe mutation (K595N and M596L) and human PS1 with deletion of exon 9 co-integrated in the same transgene [22]. The mice were backcrossed for nine generations to C57BL/6 J mice. APP/PS1dE9 mice develop progressive amyloid pathology beginning as early as 4 months of age and cognitive deficits around 12 months of age [23]. The housing conditions (National Animal Center, Kuopio, Finland) were controlled (temperature +22 °C, light from 07:00 to 19:00; humidity 50-60%), and fresh food and water were freely available. The experiments were conducted according to the Council of Europe (Directive 86/609) and Finnish guidelines, and approved by the State Provincial Office of Eastern Finland.

**Figure 1:**
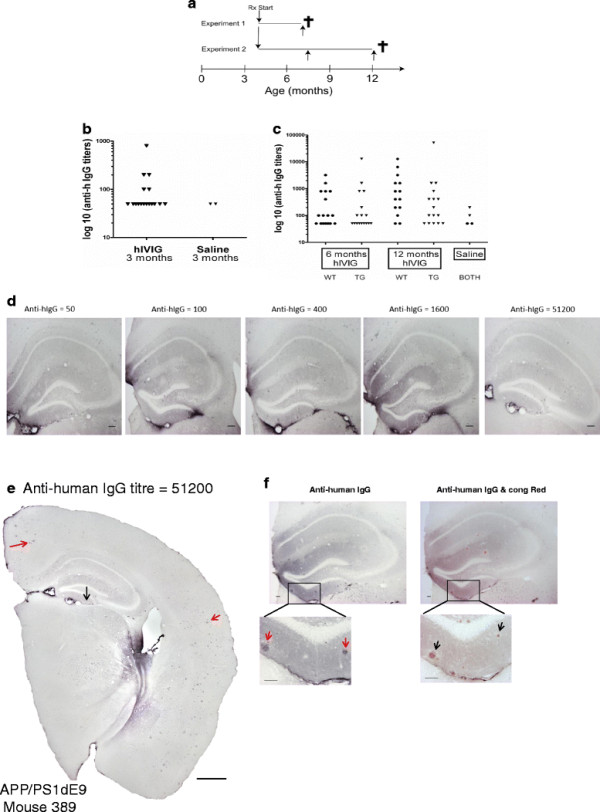
**Experiment timeline and anti-human IgG tires.** (**A**) Four-month-old APdE9 transgenic mice and their wild-type littermates received once weekly intraperitoneal injection of either hIVIG (1 g/kg) or saline for 3 months (Experiment 1) or for 8 months (Experiment 2). Mice were sacrificed one week after the last injection and brain samples were collected. In Experiment 2, blood samples were also collected at 7.5 months (upward arrows). (**B**) Anti-human IgG titers developed by APdE9 mice treated for 3 months with hIVIG (*n* = 17) compared to saline (*n* = 2). Sera were collected at the end of treatment period and anti-human IgG titers were estimated by ELISA. Titers are expressed in log10 scale and titers only > 200 were considered significant. (**C**) Anti-human IgG titers developed at 6 and 12 month’s time point by 4-month-old female APdE9 (*n* = 16) and wild-type (*n* = 16) mice, treated for 8 months with hIVIG compared to saline (*n* = 4; one for each genotype at 6 and 12 months of age). Titers > 200 were considered significant. Circles and triangles represent wild and APdE9 mice, respectively. It is evident from (**B**) and (**C**) that not all mice developed significant anti-human IgG titers. Moreover, at terminal sampling point anti-human IgG titers were highly variable irrespective of genotype or treatment duration. (**D**) Brain sections stained with anti-human IgG antibody and the corresponding anti-human IgG titres in sera as measured by ELISA are shown here. Notice similar patterns of human IgG immunopositivity in hippocampus as we proceed from titres of 50 to as high as 51,200. Scale bar denotes 100 μm. (**E**) Coronal brain section immunostained with anti-human IgG antibody. The full extent of human IgG penetration in this mouse 389 with highest anti-human IgG titres is displayed. Scale bar denotes 500 μm. Black and red arrows highlight hippocampal and cortical human IgG immunopositivity, respectively. (**F**) One of the sections from a pair of serial sections is stained with anti-human IgG antibody and the other with double stain for anti-human IgG/congo red. Brain penetration of hIVIG is clearly evident from the presence of numerous hIgG deposits in the hippocampus (red arrows). These hIgG deposits are seen decorating congo red positive amyloid deposits (black arrows). Scale bar denotes 50 μm.

We ran two experiments with treatment durations of 3 months and 8 months, beginning at the age of 4 months (Figure 1A). Mice were randomly assigned to either hIVIG (Gammagard Liquid, Baxter A/G, Austria; *n* = 16) or saline (10 mL/kg; *n* = 15) treatments, and received weekly intraperitoneal injections. The dose (1 g/kg) and treatment schedule was chosen based on our initial pilot studies. Throughout the treatment period mice were housed individually in cages. Mice were regularly assessed for weight loss, signs of pain, and distress due to treatments. Apart from tenderness and heightened tactile sensitivity around the site of injection, there were no other untoward behavioral or pathological consequences of chronic hIVIG treatment. A week after the last hIVIG injection, mice were euthanized with an overdose of pentobarbiturate-chloralhydrate injection. Terminal blood sample was collected with a cardiac puncture for assays of serum Aβ and anti-human IgG levels. Mice were then transcardially perfused with ice-cold saline. Brains were rapidly removed, subsequently one hemibrain was dissected on ice into three cortical (frontal, parietal, temporal) blocks and one hippocampal block, and snap frozen in liquid nitrogen. The other hemibrain was immersion fixed in 4% paraformaldehyde solution for 4 h followed by 30% sucrose overnight. Fresh frozen brains samples were stored at -70°C until used, whereas fixed hemibrains were stored in anti-freeze at -20°C for later immunohistology.

### **Histochemistry**

Coronal sections of 35 μm were cut with a freezing slide microtome. Following systematic random sampling scheme, four free-floating sections 400 μm apart from each other, starting from septal hippocampus (-1.3 mm from bregma) were stained with primary antibodies 6E10 (1:2,000; Senetek), WO2 (1:30,000; Genetics) for Aβ, and with CD45 (1:1,000; Serotec), CD68 (1:5,000; Serotec), Iba-1 (1:5,000;Wako) for microglia. Astrocytes were stained with GFAP primary antibody (1:1,000; Sigma). Similarly, six to seven sections each 210 μm apart starting from septal hippocampus (-1.3 mm from bregma) were stained with doublecortin antibody (1:1,000; Santa Cruz) to visualize newly-born neurons. Corresponding biotinylated anti-rat, anti-mouse, anti-rabbit, and anti-goat secondary antibodies (1:500) were used followed by incubation with HRP labeled Streptavidin. Color was developed by reacting nickel diaminobenzidine with hydrogen peroxide. Reaction was stopped with phosphate buffer and sections were then mounted, cleared in xylene, and coverslipped. For double fluorescent stainings, sections were sequentially incubated with CD45 followed by Iba-1 primary antibodies. Tyramide signal amplification kit (Molecular Probes) was used for visualizing CD45 with cy-3 label, whereas Alexa Fluor488 conjugated secondary antibody (1:400; Molecular Probes) was used for Iba-1 staining. Anti-human IgG immunostaining were performed as described in our earlier report [21]. For Congo red staining, sections mounted on slides were incubated in saturated alcoholic alkaline NaCl solution for 20 min, followed by incubation in alkaline solution of 0.2% alcoholic Congo red solution. Slides were then washed, dehydrated, cleared in xylene, and coverslipped.

### **Image acquisition and analysis**

Images (2074 × 2074) from hippocampi were acquired at 2× (Plan N2×/0.06) objective using an upright optical microscope (OLYMPUS BX40) with Olympus optical DP50 camera. A flat field image was also acquired in order to correct uneven illumination. Immunopositive areas were quantified using Photoshop CS3 Extended version 10 software (Adobe Systems Incorporated, San Jose, CA, USA). Briefly, hippocampus (the region of interest) was outlined and the immunopositive areas were thresholded for measurements and reported as mean percent immunopositive area for each brain. For double-stained fluorescent sections, images were acquired with confocal Zeiss LSM 700 microscope. Eight bit images stacks (1024 × 1024) were taken with Plan-Apochromat 40×/1.3 oil DIC M27 objective. Individual pixel size in x-y direction was 0.31 μm and 0.41 to 0.49 μm along the z-axis. Subsequent image analysis was done by Image J software. After background subtraction, individual microglial cell stacks were outlined and separated in x-, y-, and z-planes. Each stack was then split into its constituent green, red, and blue channels. A simple Image J macro then applied a threshold value for red and green channels, and created selections for measurements of integrated densities, volume, and areas. Maximum intensity projections of microglial cell stacks were then used to measure circularity.

### **Assay for anti-human IgG antibodies**

Microtiter plates (PolySorp, Nunc) were coated with Gammagard Liquid (Baxter AG, Austria) diluted to 1 μg/mL with coating buffer containing NaHCO_3_ (0.1 M; Merck) and Na_2_CO_3_ (0.1 M; Merck), adjusted to pH9.6. After overnight incubation and washing with a buffer of Dulbecco’s PBS (Gibco Life Technologies) supplemented with Tween20 (0.05%; Merck), the plates were blocked with the same buffer plus 1% BSA for 2 h at room temperature. After further washing, dilutions of serum samples and controls were added to the plates and incubated for 2 h at room temperature. Serum samples and the negative control (serum pooled from untreated mice) were pre-diluted at 1:50 in the same buffer but with 0.5% BSA and further serially diluted by a factor of 2. Purified mouse anti-human IgG Fc (Jackson) was pre-diluted 1:250,000 and further serially diluted by a factor of 2 for the positive control of the assay. After washings, the plates were incubated with HRP-conjugated goat anti-murine IgG (1:2,000; Southern Biotech) for 1 h at room temperature. Further washing and incubation with enzyme substrate (substrate buffer: Na2HPO4.2H2O (0.1 M; Merck), C6H8O7.H_2_O (0.05 M; Merck), pH5.0, *o*-phenylenediamine-dihydrochloride (1 mg/mL; Sigma), and H_2_O_2_ (0.03%; Merck)) for 30 min at room temperature produced a color reaction. The reaction was stopped with H_2_SO_4_ (2 N; Merck) and the color intensity was measured with an ELISA plate reader (Synergy BioTek) set at a 492-nm wavelength (wavelength correction set to 630 nm). All samples where the difference between the samples’ OD and the blank OD was ≥ 0.3 were considered positive. The highest dilution to show a positive result was specified as the titer.

### **ELISA assays of amyloid-β**

For estimating serum Aβ40 levels, serum samples were diluted at 1:3 with diluting buffer in accordance to the instructions of commercial high sensitivity ELISA kit (The Genetics Company, Switzerland). Standards or diluted samples and antibody conjugate comprising of a detection antibody were applied to the 96-well microtiter plates precoated with capture antibody. Following overnight incubations at 4 °C and washings, biotin-streptavidin enzyme conjugate was incubated at room temperature for 30 min. A further incubation with enzyme substrate at room temperature for 30 min gave a colored reaction product. After stopping the reaction, color intensity was measured with a microtiter ELISA plate reader (Labsystem Multiskan MS) set at 450 nm wavelength. Standard curve was prepared and sample concentrations of Aβ40 were extracted from standard curve and expressed as pg per mL of serum.

Levels of soluble and insoluble Aβ40 and Aβ42 were determined from the hippocampus block. The tissue was weighed and homogenized in 10× volume of Dulbecco’s PBS buffer (SIGMA), containing complete inhibitory mixture (Roche Diagnostics, Germany). Samples were centrifuged at 45,000 rpm (Beckman Ultrafuge) for 2 h at 4 °C. Supernatant was diluted at 1:2 and used to analyze soluble levels of Aβ40 and Aβ42. The remaining pellet was resuspended in 5 M guanidine-HCl/50 mM Tris·HCl, pH 8.0 and mixed on a shaker for 3 h at RT. Samples were then diluted at 1:50 with reaction buffer (Dulbecco’s PBS with 5% BSA, 0.03% tween-20, supplemented with protease inhibitor cocktail) and centrifuged at 16,000 × g for 20 min at 4 °C. Decanted supernantant is further diluted at 1:400 with dilution buffer. Diluted samples were then used to analyze insoluble Aβ40 and42 species. Aβ40 and Aβ42 levels were estimated using ELISA kits (Biosource International) in accordance to manufacturer’s instructions. Aβ40 andAβ42 levels were standardized to tissue weight and expressed as picograms of Aβ per gram ± SEM.

### **Real-time PCR**

Total RNA was extracted from frozen frontal cortices by TRIzol reagent (Invitrogen) according to the manufacturer’s instructions. RNA concentration and purity was measured with Nanodrop 1,000 spectrophotometer (Thermo Fisher Scientific). cDNA was synthesized from 500 ng of total RNA using random hexamer primers (Promega) as a template and Maxima reverse transcriptase (all reagents from Fermentas). The relative expression levels of mRNA encoding mouse TNF-α and IL-1β were measured according to manufacturer's protocol by quantitative RT-PCR (StepOnePlus; Applied Biosystems) by using specific assays-on-demand (Applied Biosystems) target mixes. The expression levels were normalized to ribosomal RNA and presented as fold change in the expression ± SEM.

### **Statistical analysis**

All data are given as mean ± SEM. For comparing means of two treatment groups, two independent sample student’s *t*-test was employed. For testing genotype and treatment interaction, two-way ANOVA was employed. For non-parametric data, Mann–Whitney *U* test was employed to compare treatment medians. Two-step cluster analysis was used to segregate microglia. All statistical analysis were performed by GraphPad Prism version 5.03 for Windows (GraphPad Software, La Jolla, CA, USA, http://www.graphpad.com) and IBM SPSS statistics version 19 for Windows (USA). Statistical significance was set at *P* < 0.05.

## **Results**

### **Development of anti-human IgG antibodies due hIVIG treatment in APdE9 mice**

We treated APdE9 mice with hIVIG at 1 g/kg i.p. once weekly starting at the age of 4 months, either for 3 months or 8 months (Figure 1A). As expected, treatment resulted in development of neutralizing antibodies. In the 3-month study, we noticed that by the end of the treatment all mice except only three had anti-human IgG titres below 200. This observation led us to initially conclude that mice will eventually develop immune tolerance. However, our 8-month study clearly showed that this was not the case. Anti-human IgG titres remained highly variable irrespective of genotype (Figure 1 B and C). This is in agreement with a recent report describing murine anti-human antibody response of natural human antibodies in a mouse model of Alzheimer’s disease [24]. However, it was evident that brain penetration of hIVIG was not affected by the magnitude of anti-human IgG titres. In Figure 1D one can clearly see positive background immunostaining for human IgG in the hippocampus and even abutting putative plaques to a similar extent in mice with low *vs.* high titres of neutralizing antibodies. Also, the decoration of amyloid plaques by human IgG in both cortex and hippocampus of a mouse (Figure 1E) that had the highest anti-human IgG titres indicates that brain penetration of hIVIG is not limited by the development of anti-human antibodies. Lastly, stippled patterns of aggregated material covered with human IgG immunoreactivity is specific to amyloid plaques as clearly demonstrated in sections double stained with both anti-human IgG immunostain and congo red histological stain (Figure 1F).

To further assess the contribution of neutralizing antibodies to the results, we correlated the main measured outcome parameters with anti-human antibodies. Serum Aβ40 (r = 0.035, *P* = 0.91), hippocampal soluble Aβ40 (r = -0.11, *P* = 0.68), hippocampal CD45 immunoreactivity (r = -0.29, *P* = 0.27), TNF-α (r = 0.01, *P* = 0.98) and Doublecortin positive cells in the dentate gyrus (r = -0.11, *P* = 0.76) all yielded statistically non-significant correlations. Finally, among the 16 mice that received hIVIG only seven mice produced significant titres of neutralizing antibodies (titres >200). After removing data from these mice, the outcome of the study remained unchanged. These data are presented in corresponding sections below.

### **Effects of hIVIG treatment on amyloid pathology in APdE9 mice**

**Figure 2:**
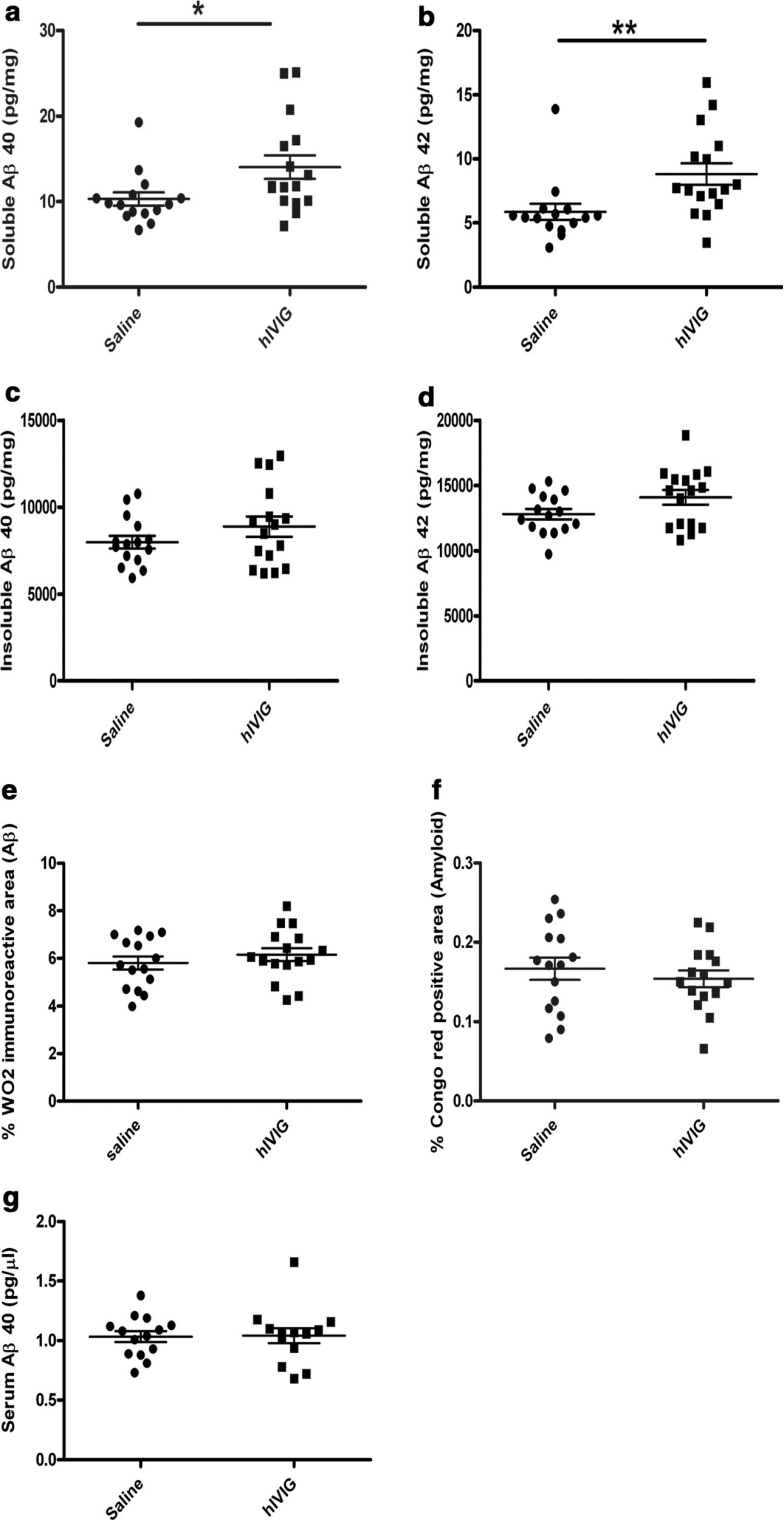
**Effects of chronic (8 months) hIVIG treatment on amyloid pathology in APdE9 mice.** Brain samples were analyzed from 16 hIVIG treated and 15 saline treated mice. Chronic hIVIG treatment elevated soluble Aβ40 (**A**) and Aβ42 (**B**) levels in the hippocampus. In contrast, levels of hippocampal insoluble Aβ40 (**C**) and Aβ42 (**D**) levels did not change significantly. There was no significant treatment effect on amyloid load (relative amyloid plaque area) in the hippocampus as visualized by WO2 antibody (**E**) or on dense core amyloid plaque area (**F**). Serum Aβ40 levels (**G**) in saline (*n* = 14) and hIVIG (*n* = 14) treated mice did not differ, either. All data are given as mean ± SEM, * *P* < 0.05, ** *P* < 0.01 as calculated by student’s *t*-test.

Transgenic APdE9 mice treated for 3 months showed no treatment effects compared to the saline group on either soluble or insoluble Aβ40 or Aβ42 levels in the hippocampus or on serum Aβ40 levels (Table 1). Furthermore, amyloid plaque burden in the hippocampus as visualized by 6E10 antibody and Congo red stain did not differ between saline and hIVIG treated mice. Similarly, quantification of CD45 immunostaining revealed no treatment effects (Table 1). In contrast, APdE9 mice receiving the same weekly hIVIG treatment for 8 months demonstrated a significant elevation of soluble Aβ40 (t_29_ = 2.3, *P* = 0.03) and Aβ42 (t_29_ = 2.8, *P* = 0.009) levels in the hippocampus (Figure 2A and B). After removing data from mice that produced significant titres of anti-human antibodies, elevation of soluble Aβ42 levels still remained significant (t_22_ = 2.2, *P* = 0.038). Insoluble Aβ40 and Aβ42 levels were also elevated but did not reach statistical significance (*P* > 0.21; Figure 2C and D). In agreement with this, immunohistochemical analysis of amyloid plaques by W02 antibody (*P* = 0.37) and by Congo red staining (*P* = 0.47) revealed no treatment differences (Figure 2E and F). In addition, serum Aβ40 levels did not differ between the treatment groups (Figure 2G; *P* = 0.92), and brain levels of Aβ40 and Aβ42 showed no correlation with serum Aβ40 levels (r = 0.29, *P* = 0.31; r = 0.32, *P* = 0.26, respectively). These data indicate a limited role of peripheral sink as one of the mechanisms whereby hIVIG can influence brain soluble Aβ levels.

**Table 1:** Amyloid pathology and microglial reactivity after 3 months of hIVIG treatment in APdE9 mice

**Measured parameter**	**Treatments**		***P*value**
	**Saline**	**hIVIG**	
Serum Aβ40 (pg/μL)	0.56 ± 0.07	0.64 ± 0.07	0.67
Soluble Aβ42 (pg/mg)	0.36 ± 0.12	0.32 ± 0.05	0.74
Insoluble Aβ42 (ng/μL)	2.41 ± 0.25	2.43 ± 0.16	0.93
Aβ deposits (% 6E10 immunopositive area)	1.26 ± 0.08	1.20 ± 0.09	0.66
Fibrillar Aβ (% Congo red positive area)	0.10 ± 0.01	0.12 ± 0.01	0.12
Microglia (% CD45 immunopositive area)	0.84 ± 0.06	0.83 ± 0.06	0.90

Four-month-old APP/PS1dE9 mice were treated with once weekly intraperitoneal injection of either hIVIG (1 g/kg; *n* =16) or saline (*n* = 14) for 3 months. At the end of the treatment period, brains were extracted to assess amyloid and microglial pathology. Serum Aβ40, soluble and insoluble Aβ42 were measured by ELISA. Aβ deposits and microglial activity were quantitatively assessed from immunostainings whereas fibrillar amyloid was assessed by quantifying Congo red histological stain. Data are represented as mean ± SEM. Student’s *t-*test was employed to find treatment differences and *P* < 0.05 was considered statistically significant.

### **Chronic hIVIG treatment suppresses microglia**

**Figure 3:**
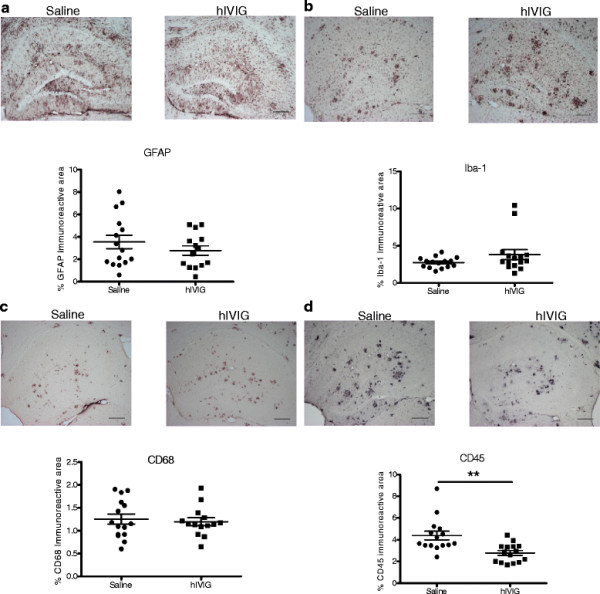
**Effects of chronic (8 months) hIVIG treatment on glial pathology in APdE9 mice.** Immunostainings specific for astrocytes and microglial cells were performed on free-floating brain sections. Panel shows representative images from hippocampi of saline (*n* = 15) and hIVIG (*n* = 14-16) treated mice. (**A**) Astrocyte reactivity measured as GFAP immunoreactivity. (**B**) Microglial reactivity as measured as Iba-1 immunoreactivity. (**C**) Lysosomal marker CD68 immunoreativity. (**D**) Microglial activation as measured by CD45 immunoreactivity showed significant differences between the treatments. Scale bars represent 200 μm. Data are given as mean ± SEM. * *P* < 0.05, Student’s *t*-test. Scale bar represents 200 μm.

We next went on to assess the nature of neuroinflammation associated with amyloid plaques in APdE9 mice treated chronically for 8 months. Astrocyte reactivity as measured by GFAP immunopositive area in hippocampi did not differ between the treatment groups (Figure 3A). Microglia/tissue macrophage reactivity in saline and hIVIG treated mice was assessed by three markers: Iba-1a, CD68, and CD45. Iba-1a is a structural marker for all microglia, while CD45 is expressed more intensively in infiltrating leukocytes compared to resident microglia. Furthermore, increased expression of CD45 on resident microglia indicates a phenotype of microglia activated primarily by Aβ deposits. CD68 is a lysosomal marker indicative of phagocytosis in microglia/macrophages [2,25-27]. Whereas Iba-1a and CD68 markers revealed no significant treatment effects (Figure 3B and C), CD45 immunoreactivity was significantly decreased (approximately 30%) in hIVIG treated mice (Figure 3D). After removing data from mice that produced significant titres of anti-human antibodies, the main outcome, suppression of CD45 still remained significant (t_22_ = 3.0, *P* = 0.0064). Moreover, this suppression correlated inversely with elevated levels of soluble Aβ in the hippocampus, indicating that these two effects of chronic hIVIG treatment were interrelated (solubleAβ42: r = -0.43, *P* = 0.02).

### **Chronic hIVIG treatment induces subtype specific changes in microglia**

**Figure 4:**
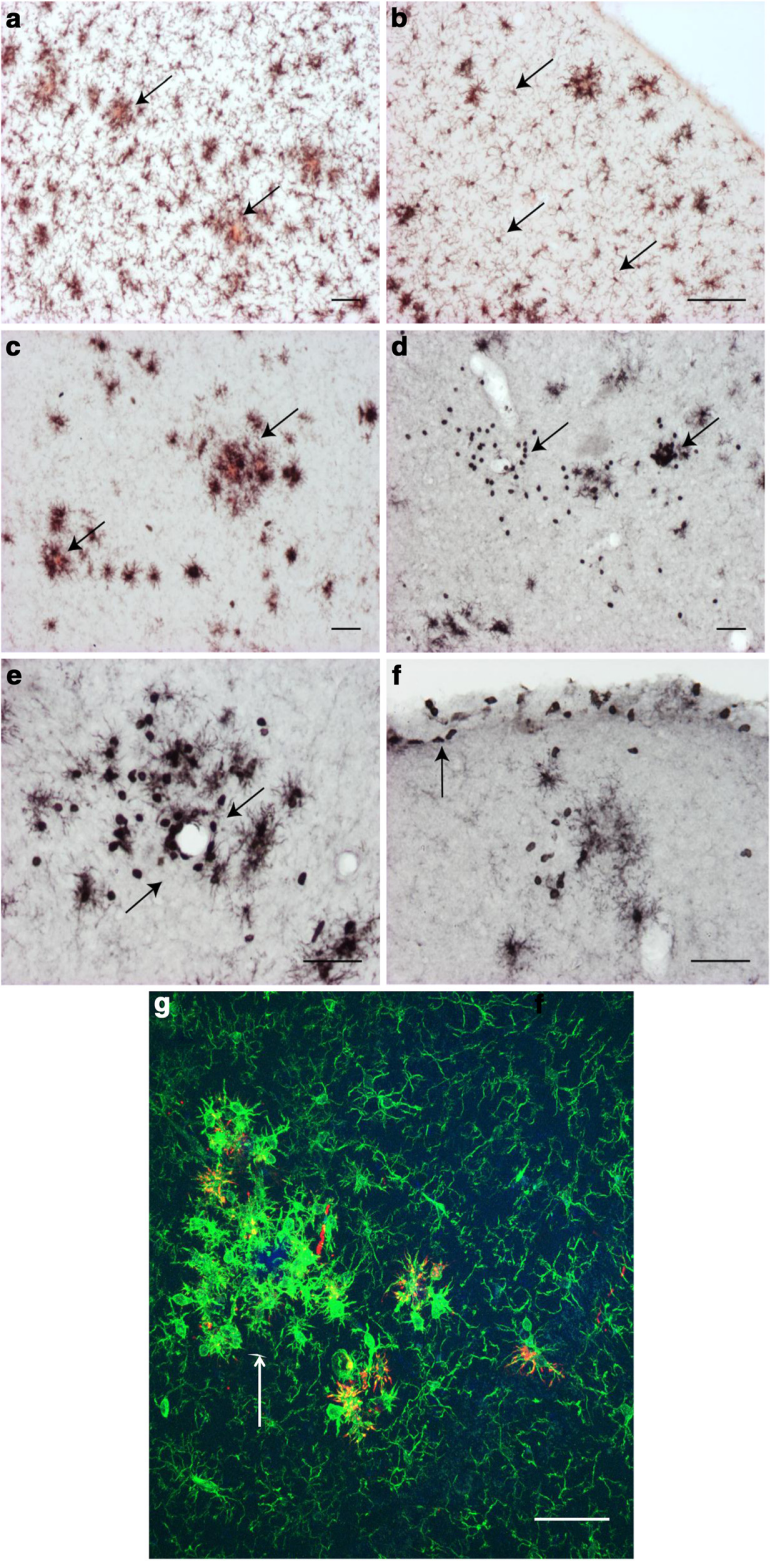
**Iba-1 and CD45 markers immunostain different phenotypes of microglial cells.** Immunohistochemical detection with Iba-1 antibody identified microglia both near and away from amyloid deposits. (**A**) Activated microglia near and around Congo red positive amyloid deposits (arrows) show increased Iba-1 expression and possess larger cell bodies with short thick processes. Typically, these activated microglia cluster together forming a cordon around amyloid deposits. (**B**) Microglia away from or in between amyloid deposits (arrows) differ in morphology from the previous type with respect to their smaller cell bodies and long thinner processes. These quiescent microglia typically maintain distance from each other and are actively receiving inputs about their respective microenvironments [29] (**C**) The microglial marker CD 45 clearly identifies only activated microglia near amyloid deposits and possesses larger cell bodies with short thick processes. CD45 antibody stained no microglia away from or in between Aβ deposits. (**D**) CD45 also identified many dark intensively stained round cells (arrows) which were often found isolated or in groups inside brain parenchyma or near blood vessels (**E**) or meninges (**F**). (**G**) Phenotypes marked by both Iba-1 and CD45 can be seen together in a double fluorescent immunostaining. Maximun intensity projection of a confocal Z-stack revealed distinct Iba-1 (green) and CD45 (red) positive cell populations pointing to simultaneously co-existing different morphological phenotypes of activated and resting microglia. Intensively stained iba-1 positive microglia (green) are seen as clumps (arrow) around a putative amyloid deposit. Some but not all microglia (red) in clumps were also CD45 positive, thus producing a yellow signal indicative of co-localization. In contrast, quiescent microglia away from putative amyloid deposits showed less intense Iba-1 (green) signal. This way it is feasible to simultaneously identify and quantify microglial cells based on their morphological (circularity) and biochemical (Iba-1 and CD45 expression) attributes. Scale bars represent 500 μm except for the confocal fluorescent image where it represents 50 μm.

A closer examination of Iba-1a and CD45 immunopositive cells revealed differences in their morphology and location with regard to amyloid plaques. Iba-1a immunopositive cells with variable staining intensity could be found both in the immediate vicinity of amyloid plaques (Figure 4A) and in areas free of plaques (Figure 4B). In contrast, CD45 immunoreactivity was increased specifically near amyloid plaques (Figure 4C). In addition, we found small round, strongly CD45 positive cells randomly distributed in the brain parenchyma (Figure 4D) and in scattered dense clusters around brain blood vessels (Figure 4E) and meninges (Figure 4F). It is reasonable to hypothesize that these different populations of Iba-1a or CD45 positive putative microglia and tissue macrophages respond differentially to hIVIG treatment, resulting in the observed decrease in the total CD45 signal.

**Figure 5:**
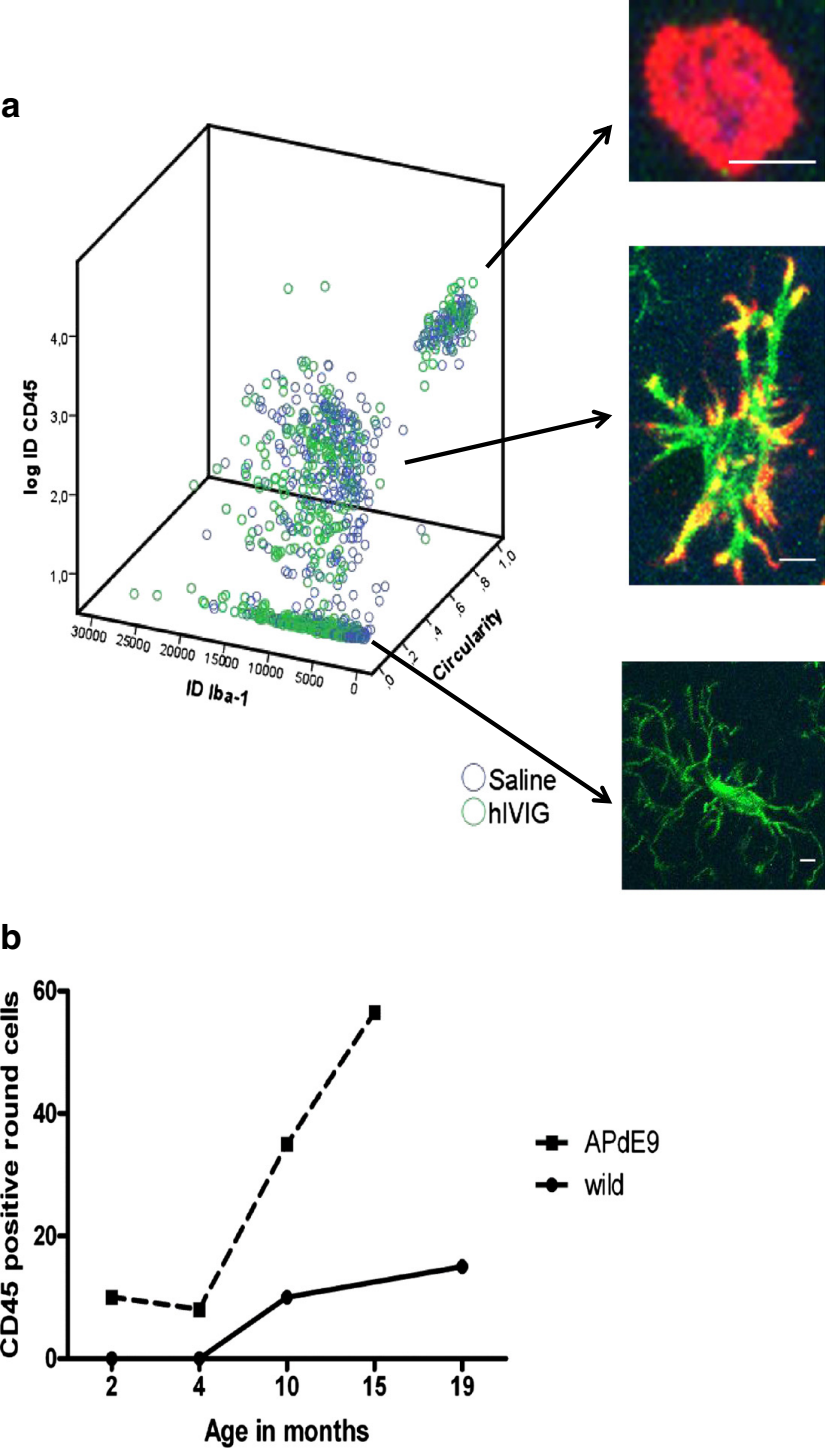
**Chronic hIVIG treatment produces cell type specific changes in microglia.** In order to assess whether chronic hIVIG treatment had differential effects on morphologically discernible phenotypes of microglia, we analyzed expression of Iba-1 and CD45 on each individual microglial cell along its entire X-Y-Z planes. Coronal sections of 35 μm were double stained with Iba-1 and CD45 primary antibodies followed by attaching fluorescent secondary antibodies. We then acquired confocal Z-stack images from hippocampi of both saline and hIVIG treated mice. After identifying and segregating the X-Y-Z boundaries of each microglial cell, a simple Image J macro measured the area, volume, and integrated densities for Iba-1 and CD45 markers across its Z-axis. Circularity was measured using maximum intensity projection image for each of the microglial stack. We analyzed 955 randomly chosen microglial cells spread across saline (*n* = 6) and hIVIG (*n* = 6) treated groups. (**A**) A 3-D scatter plot of CD45 and Iba-1 integrated intensities, and circularity for each microglia cell type. Green circles represent the hIVIG group and blue circles the saline group. Both visually and statistically, one can easily discern three clusters of cells which we designated as type A (*n* = 134; 14%), type B (*n* = 386; 40.4%), and type C (*n* = 417; 43.6%). The right panels illustrate representative cells of each type. Round morphology and high expression of CD45 alone characterizes type A cells. Type B cells features include ramified morphology with comparable expression of both CD45 and Iba-1 markers, whereas type C cells exhibit a ramified morphology but express predominantly Iba-1 marker. Scale bar = 5 μm. (**B**) The number of CD45 positive round cells encountered in brain parenchyma (cortex and hippocampus) of APdE9 *vs.* wild-type mice at 2, 4, 10, 15, and 19 months of age (*n* = 2, at each age point). APdE9 mice exhibit a much more conspicuous age-dependent increase in the number of CD45 positive round cells than their wild-type littermates.

In order to separate treatment effects in different cell populations, we measured individual microglial cells on confocal Z-stacks for their CD45 and Iba-1 intensities (Figure 4G). The mean integrated intensities of CD45 and Iba-1 markers across the entire Z-axis of each microglia were plotted against the circularity (morphological parameter). This 3-D scatter plot visually identified three different sub-populations of microglia as shown in Figure 5. Statistically, two-step cluster analysis as well revealed three different clusters of cells with an average silhouette equal to 0.7. In addition, the nonparametric Kruskal-Wallis H-test rejected the null hypothesis (*P* < 0.001) that there are no sub-populations of microglia. We call these three clusters of cells simply as type A, B, and C (Figure 5A). Type A cells seen in the plot have a round soma with no processes, and exhibit very high CD45 immunostaining intensity but negligible Iba-1a staining. These cells correspond to round cells illustrated in Figure 4D and E). Their number increases dramatically from 4 to 12 months of age, and much faster in APdE9 mice than in wild-type control mice (Figure 5B). *Prima facie*, they might be infiltrating immune cells, although it is difficult to ascertain their exact peripheral origin in this particular experimental setup. Chronic hIVIG treatment did not significantly affect CD45 expression in type A cells (U = 2,117, *P* = 0.99, Mann–Whitney *U* test). Type B cells are characterized by large oval cell bodies and thick branching processes, and exhibit substantial CD45 and Iba-1 signal. These cells are located in the immediate microenvironment of amyloid plaques and likely represent activated microglia around Aβ deposits [28]. Mann–Whitney *U*-test revealed significantly reduced expression of CD45 (U = 15,605, *P* < 0.02) and elevated expression of Iba-1 (U = 13,972, *P* < 0.0001) in type B cells as a result of chronic hIVIG treatment. Lastly, type C cells show much more variable cell morphology. The cell bodies are relatively small and the processes much thinner than in type B cells. Type C cells exhibit substantial Iba-1a expression but practically no CD45 signal. These cells are found equally often around Aβ deposits and in plaque-free tissue. Those away from the plaques likely represent cells referred to in the literature as quiescent microglia [25,28,29]. Iba-1a expression was significantly elevated in type C cells as a consequence of chronic hIVIG treatment (U = 12,795, *P* = 0.0001). In hIVIG treated mice type C microglial cells located away from amyloid deposits were with comparatively longer and highly ramified processes than those seen in saline treated mice. Collectively, these observations indicate that chronic hIVIG treatment brings about specific changes in subpopulations of microglia/macrophages.

### **Chronic hIVIG treatment suppresses TNF-α gene expression**

**Figure 6:**
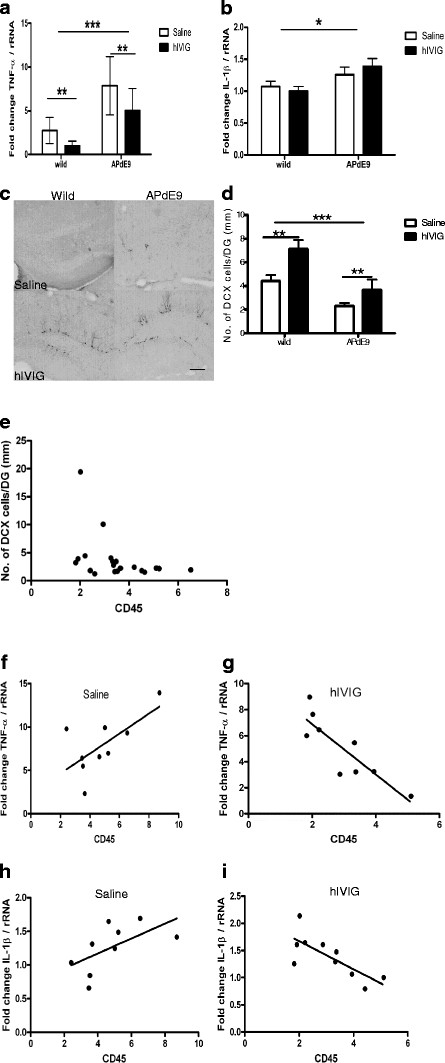
**Chronic hIVIG treatment decreases TNF-α gene expression and boosts neurogenesis.** Frozen tissue samples from frontal cortices of APdE9 and wild-type mice (*n* = 8-10) in saline and hIVIG groups were examined by quantitative RT-PCR for relative expression levels of TNF-α (A) and IL-1β (B) mRNA (in comparison to expression levels of ribosomal RNA). Genotype and treatments effects were tested using two-way ANOVA. (**A**) For TNF-α after removal of an outlier in hIVIG treated APdE9 group, significant genotype (***, *P* < 0.01), and treatment effects (***, *P* < 0.01) were noticed albeit without any interaction. (**B**) IL-β showed only genotype differences (*, *P* < 0.05). (**C**) Representative images from doublecortin stained hippocampal sections of wild-type and APdE9 transgenic mice treated for 8 months with saline or hIVIG. Scale bar represent 100 μm. (**D**) The doublecortin data are presented as mean ± SEM (*n* = 10 mice in each group). After removal of an outlier in the hIVIG treated APdE9 group, significant genotype (***, *P* < 0.01) and treatment effects (**, *P* < 0.01) emerged, with no significant interaction. (**E**) CD45 positive microglia also showed a significant negative correlation with the number of doublecortin positive cells in the dentate gyrus (Spearman r = -0.50, *P* = 0.02). Spearman correlation coefficient was used here to minimize the effect of two outliers. (**F**) Saline treated APdE9 mice showed a trend towards positive correlation between CD45 microglia and TNF-α (Pearson r = 0.66; *P* = 0.05). (**G**) A significant negative correlation was observed between CD45 microglia and TNF-α expression in hIVIG treated mice (Pearson r = -0.86; *P* = 0.003). (**H**) Similarly, relative levels of IL-β expression showed a trend for positive correlation (Pearson r = 0.59; *P* = 0.09) in saline treated mice, whereas hIVIG treatment (**I**) brought about a significant inverse correlation (Pearson r = -0.76; *P* = 0.01) between these two variables.

We further addressed the question of whether hIVIG effect on subpopulations of microglia/macrophages is reflected in gene expression of pro-inflammatory cytokines TNFα and IL-1β. Two-way ANOVA analysis of gene expression profiles of these cytokines revealed significant genotype (F_1,33_ = 34.3, *P* < 0.001) and treatment (*P* = 0.007) effects for TNF-α, but no significant interaction between these factors (*P* = 0.50; Figure 6A). As for IL-1β, we noticed a significant genotype effect (F_1,34_ = 7.2, *P* = 0.01) but no treatment effect (*P* = 0.79) or genotype by treatment interaction (*P* = 0.36; Figure 6 B). Interestingly, in saline treated APdE9 mice there was a trend towards positive correlation between TNF-α mRNA levels and CD45 positive microglia (immunopositive area), while a significant negative correlation was found between TNF-α expression and CD45 microglia in hIVIG treated mice (Figure 6E and F). Similar opposite correlations were also observed between the treatment groups for IL-1β (Figure 6G and H). These findings suggest that chronic neuroinflammation around amyloid plaques in APdE9 mice is associated with a general increase in pro-inflammatory cytokines levels, while hIVIG specifically suppresses some cytokines such as TNFα.

### **Chronic hIVIG treatment augments neurogenesis**

Finally, since involvement of microglia has been suggested in hippocampal neurogenesis [30-35], we wanted to assess whether chronic hIVIG treatment with selective effects on some microglia/macrophage subpopulations influences hippocampal neurogenesis. To this end, we quantified doublecortin immunoreactivity in the dentate gyrus (DG) of the hippocampus (where adult neurogenesis is observed specifically in the subgranular zone). Consistent with our previous unpublished findings, barely any neurogenesis was present in the DG of saline treated mice at the age of 12 months. However, a substantial number of doublecortin immunopositive neurons could be observed in the DG of hIVIG treated mice (Figure 6C and D). After removal of one outlier in the hIVIG group, two-way ANOVA revealed a significant main effects of treatment (F_1,34_ = 10.0, *P* = 0.003) and genotype (F_1,34_ = 18.9, *P* = 0.0001) but no genotype by treatment interaction (*P* = 0.30). Furthermore, CD45 positive microglia (immunopositive area) also showed a significant negative correlation with the number of doublecortin cells in the DG (Figure 6 I). These findings indicate that chronic hIVIG treatment may preserve the capacity of adult neurogenesis until a late age even in the presence of amyloid pathology, and that this effect may also be related to immunomodulation.

## **Discussion**

Currently, human intravenous immunoglobulins (hIVIG) are being tested in a Phase-3 clinical trial in mild to moderate AD patients [36]. However, the mechanism of action for hIVIG still remains elusive. In order to investigate chronic *in vivo* effects of hIVIG, we treated APP/PS1dE9 transgenic mice with weekly injections for 3 months and 8 months in separate studies. The shorter, 3-month treatment duration, did not bring about any noticeable changes in amyloid or microglial parameters. However, the longer, 8-month treatment led to significant elevation of soluble hippocampal Aβ levels with no significant changes in plaque-associated Aβ. Chronic treatment also resulted in elevation of Iba-1 but suppressed CD45 marker in a subset of microglia/tissue macrophages and reduced pro-inflammatory cytokine expression. In addition, the chronic hIVIG treatment augmented neurogenesis, the extent of which further correlated with changes in the CD45 marker. These findings suggest that long-term hIVIG treatment alters the brain neuroinflammatory response towards beneficial direction. In contrast to our previous report, long-term hIVIG treatment in APP/PS1 mice resulted in development of anti-human antibodies. However, immunohistochemical presence of human IgG inside cortex and hippocampus confirmed brain penetration of hIVIG and together with re-examination of statistical data after excluding mice that developed neutralizing antibodies, indicate that neutralizing anti-human antibodies made only a minor contribution to the present results.

We used the most concentrated hIVIG preparation available (1 g/kg) to maximize the dose of putative anti-Aβ antibodies in this study. On the other hand, in a human study on patients with mild AD, a lower dose (0.4 g/kg) of hIVIG was found to be associated with higher MMSE scores than the high 1 g/kg dose, which points to an inverted dose–response for *in vivo* hIVIG effects [20]. Therefore, it appears logical to expect more robust effects at a lower dose of hIVIG also in an AD mouse model. However, our own studies in APP/PS1dE9 mice with either a low dose (0.1 g/kg) or shorter treatment durations (1-3 weeks) yielded no effects on amyloid or glial pathology. Moreover, our previous studies [21] indicate that either a lower dose or a shorter treatment duration do not yield a sufficient brain penetration of hIVIG. Furthermore, the brain bioavailability of hIVIG is likely to be much higher in aged humans than in young adult mice and with intravenous administration as used in human trial one may end up with a dose comparable to intraperitoneal administration of the high dose used in this study.

Human IVIG has been reported to possess antibodies reactive not only to Aβ epitopes but also to Aβ conformation [18]. Furthermore, both epitope and conformation specific Aβ antibodies have been reported in sera from healthy humans irrespective of age or gender [37]. These antibodies present in hIVIG are hypothesized to augment the clearance of Aβ from the brains of APP/PS1dE9 mice by various established mechanisms, such as antibody mediated disaggregation of Aβ deposits, antibody mediated microglial phagocytosis of Aβ deposits, and antibody mediated efflux of Aβ protein from CNS into peripheral circulation due to peripheral sink. Notably, all these mutually exclusive mechanisms should ultimately result in decrease in brain total Aβ levels, and the peripheral sink effect in addition may lead to an increase in serum Aβ levels [38]. However, after 3 months of treatment with hIVIG we noticed no change in either brain or serum Aβ levels. This might be due to very low concentration of anti-Aβ antibodies of the total IgG (approximately 0.1%, [18]) and insufficient accumulation in the brain with a short duration of treatment. More surprisingly, we noticed an elevation of soluble Aβ levels with the present chronic 8-month treatment. The underlying mechanisms for the selective elevation of soluble Aβ levels is not clear at present, but interestingly, microglia ablation in APP transgenic mice resulted in similar changes in soluble Aβ levels [39]. Furthermore, APP transgenic mice with CD45 deficiency also exhibited elevation of soluble Aβ levels [40] and then in this study, chronic hIVIG treatment caused suppression of CD45 microglia marker by 30%, which correlated with elevated soluble Aβ levels. This may be evidence for the role of microglia in modulating the soluble levels of Aβ. Nonetheless, this observation together with no changes in insoluble (practically plaque-associated) Aβ clearly points to an inefficient disintegration of pre-existing Aβ deposits due to hIVIG treatment. Immunostainings with N-terminal specific antibodies or Congo red staining for fibrillar Aβ deposits also revealed no treatment effects. Our 8-month study had an 80% power to detect a 19% change in mean Aβ immunopositive area and 30% change in mean Congo red positive area with a significance level (alpha) of 0.05 (two-tailed). This together with no changes in CD68 immunostaining speaks against enhanced Aβ phagocytosis by microglia due to hIVIG treatment.

According to the peripheral sink hypothesis, bidirectional transfer of Aβ between CNS and periphery would lead to elevated serum Aβ levels [41]. However, irrespective of the dose used or treatment duration, we noticed no significant elevation of serum Aβ levels in our studies. Furthermore, there was no correlation between brain Aβ and serum Aβ levels. Statistically, our chronic 8-month study had an 80% power to detect a 22% change in mean serum Aβ_40_ levels with a significance level (alpha) of 0.05 (two-tailed). Furthermore, in our parallel unpublished studies no difference in amyloid plaque burden or in biochemical levels of Aβ in brain and serum was noticed after three weekly hIVIG injections in 3-month-old APP/PS1dE9 mice or after bi-weekly hIVIG treatment for 3 weeks in 15-month-old APP/PS1dE9 mice (data not shown). These findings indicate that the outcome of Aβ-related measurements was not dependent on age or amyloid plaque burden at the onset of hIVIG treatment. A recent *in vivo* study reported that natural Aβ-antibodies (Nabs-Aβ) isolated from hIVIG reduced amyloid plaque burden in 3-month-old mice but not in 12-month-old APP transgenic mice [24]. This reduction was ascribed to the peripheral sink mechanism. However, even in that study elevated plasma Aβ levels were not statistically significant outcome of the treatment, and in the absence of correlations between brain/CSF and plasma levels of Aβ, it will be difficult to acknowledge the presence of a peripheral sink mechanism. Moreover, the mere increase in plasma Aβ due to antibody treatment might also be a consequence of stabilization of Aβ-antibody complexes in plasma [42,43]. Interestingly, a recently published report on monoclonal anti-Aβ immunotherapy (Bapineuzumab) indicates that in spite of antibodies engaging brain Aβ, it may not necessarily be reflected in CSF or serum [44].

Even though we found no amyloid reduction with hIVIG, a recent *in vivo* study reported that natural Aβ-antibodies (Nabs-Aβ) isolated from hIVIG confer protection from Aβ toxicity along with beneficial effects on cognition in an APP transgenic mouse model of AD [24]. However, in that report absence of an hIVIG treated group makes it difficult to delineate the effects of constituent anti-Aβ antibodies and non-specific polyclonal IgG antibodies from those arising from extraction procedure itself. As expected, 4 weeks of treatment with natural Aβ-antibodies did not alter plaque pathology in 12-month-old mice but reduced amyloid plaque burden in 3-month-old mice. However, the plaque reduction effect in young animals did not differ significantly between low and high doses of Nabs-Aβ. In addition, a recent meeting abstract also reported no significant changes in PIB retention in brains of small number of human patients treated with hIVIG [45]. Collectively, these findings suggest that hIVIG unlikely would substantially affect brain Aβ deposition or clearance. 

After finding no evidence for amyloid lowering mechanisms for hIVIG in our study, we shifted our focus to anti-inflammatory actions of hIVIG. Anti-inflammatory properties of hIVIG are well-known and polyclonal antibodies of hIVIG are reactive to a wide array of inflammatory proteins including cytokines and chemokines [46]. Mean GFAP immunopositive area for astrocyte reactivity was lower in hIVIG treated mice than in the saline group, but failed to reach statistical significance. A change in microglia phenotype (or activation state) was indicated by increased expression of microglial markers around the Aβ microenvironment, with CD45 measuring activated microglia [47] and Iba-1 measuring both activated and quiescent microglia. In addition, the CD68 marker has been associated with phagocytosis [2]. The lack of significant changes in CD68 immunopositive microglia confirmed limited effects of hIVIG treatment on Aβ phagocytosis. Whilst 8-month chronic hIVIG treatment suppressed CD45 expression, Iba-1 expression increased in a subset of microglial cells as demonstrated by confocal analysis. The fact that different subtypes of microglia cells responded differently, strongly indicates that hIVIG has a highly specific immunomodulatory effect and not just a general suppression of the brain inflammatory reaction. Notably, we measured Iba-1 signal from individual microglial cells across their Z-axis, so there was no interference from microglia in other activation levels that could confound quantification of this signal. In contrast, with nickel-DAB developed sections, the immunopositive Iba-1 signal from microglia often are influenced by neighboring microglial cells present in various levels of activation. Thus, this approach allowed us to measure integrated intensities of more than one activation marker in a single individual microglial cell and relate this information to their morphological features (in this case, circularity). To our knowledge, this is the first study to report segregation of microglial cells based on both morphology and activation markers. A change in microglial activation as a consequence of hIVIG treatment has been reported in one previous *in vitro* study [48]. In this study, BV-2 microglia cell line displayed a more ramified morphology and increased expression of CD45 in response to hIVIG treatment. In contrast, our *in vivo* data finds decreased CD45 expression (only in Type B microglial cells) and increased Iba-1 expression (in Type B and Type C microglial cells) along with a shift towards more ramified microglia morphology in hIVIG treated mice.

After finding clues for differential activation of microglial cells, we focused further on classic inflammation regulatory actions of hIVIG. Many *in vitro* and *in vivo* studies have indicated the ability of hIVIG preparations to modulate cytokine induction and release [49-56]. In addition to direct cytokine neutralizing antibodies in hIVIG, there are also other plausible mechanisms [57-59]. Since pro-inflammatory cytokines like TNF-α and IL-1β are important contributors of neuroinflammation, there is an opportunity for hIVIG to cease, decrease, or suppress amyloid induced cytokine release by microglia. TNF-α gene expression has been reported to be increased in various transgenic mouse models of AD, including the APP/PS1dE9 mouse [60]. In our study, hIVIG treatment caused a significant reduction in TNF-α gene expression (and a similar trend in IL-1β expression). Moreover, this suppression of pro-inflammatory gene expression due to hIVIG treatment was not genotype specific as it was also observed in wild-type littermates. One would expect a straightforward correlation between the expression levels of pro-inflammatory cytokines and CD45 marker of activated microglia. Indeed, in saline treated mice, a trend towards positive correlation was found between CD45 microglial marker and TNF-α gene expression. However, chronic hIVIG treatment shifted this correlation to opposite direction, and a similar shift after hIVIG treatment was observed in the correlation between CD45 and IL-1β gene expression. This inverse relationship between pro-inflammatory cytokine and CD45 expression suggests that hIVIG treatment may change the brain immune response such that microglia may maintain a high activity despite a decrease in generalized inflammatory response mediated through pro-inflammatory cytokines. This profile of immunomodulation works against the profile associated with aging [6] and may confer neuroprotection in the AD brain. Pro-inflammatory cytokine regulatory effects of hIVIG are further supported by a report where circulating levels of TNF-α and IL-1β cytokines dropped upon hIVIG treatment in Guillian-Barré syndrome patients [61]. More detailed experiments are still needed to fully elucidate the mechanisms involved and judge the therapeutic potential of cytokine regulatory effects of hIVIG in the context of AD and other neurodegenerative pathologies.

Paucity of any previous studies pointing towards neurogenesis by hIVIG compelled us to discuss this indirectly. In fact in diverse experimental set-ups, many studies connect suppression of microglia and pro-inflammatory markers to different stages in neurogenesis [62-65]. Inflammation has been long known to be a negative regulator of neurogenesis [32,34]. Cellular mediators of neuroinflammation, astrocytes and microglia, may impact neurogenesis negatively via HPA axis mediated release of glucocorticoids that suppress neurogenesis or by decreasing neurotrophin support or by excessive release of reactive oxygen species and pro-inflammatory cytokines [66,67]. In addition, pro-inflammatory cytokines released by microglia are known to play role in *in vitro* differentiation of hippocampal progenitor cells [68]. Furthermore, in diverse animal models of disease pro-inflammatory cytokines TNF-α [34,69,70], IL-1β [71,72], IFN-α [73], and IL-6 [34,74] have been shown to suppress adult neurogenesis, while in one study blockade of IL-6 alone restored it [34]. In the context of AD, neurogenesis has been extensively studied in various transgenic Alzheimer mouse models, and Aβ itself has been shown to suppress adult neurogenesis in the hippocampus [75]. Strikingly, in our AD mouse model, chronic hIVIG treatment not only altered activation status of microglia and suppressed pro-inflammatory TNFα gene expression, but also significantly enhanced the number of doublecortin positive cells in the dentate gyrus irrespective of the genotype of mice. This effect may be selectively beneficial for aged mice in which neurogenesis is about to fade out. Moreover, the negative correlation between CD45 microglia and number of doublecortin positive cells in our study also points towards an inverse relationship between activated microglia and neurogenesis. While microglial cells activated by TNF-α and IL-6 inhibited neurogenesis, microglial cells activated by IL-4 and T-cells have also been reported to contribute to hippocampal neurogenesis [76,77]. Such alternatively activated microglia were shown to express MHCII proteins and co-localize with IGF-1, a growth factor known for neuroprotection and neurogenesis [78,79]. Therefore, modulation of microglia by hIVIG and subsequent suppression of pro-inflammatory cytokines might restore and foster hippocampal neurogenesis, thereby representing a mechanism to compensate for the loss of neurons observed during AD progression. However, it is also possible that fostering neurogenesis might be a direct exclusive property of hIVIG, irrespective of other indirect mechanisms discussed here.

Our study was designed to test amyloidocentric mechanisms for hIVIG but we clearly present evidence for hIVIG effects independent of Aβ clearance. This is the first passive immunotherapy report in AD transgenic mice that describes modulation of microglial activation, suppression of pro-inflammatory cytokine gene expression, and enhancement of neurogenesis as a consequence of hIVIG treatment independent of amyloid clearance in the brain. However, it is unclear whether these are mutually exclusive and independent effects. This combination of effects makes hIVIG a unique drug candidate that targets multiple inflammatory antigens and immunomodulatory factors associated with neurogenesis [80]. It will be interesting to investigate if mere modulation of CD45 microglial protein expression could as well change cytokine profiles and support neurogenesis. A recent report described alterations in plasma cytokines levels in mild to moderate AD patients treated chronically with hIVIG, and further that these changes correlated with clinical outcomes, suggesting that immunomodulation by cytokines may be one of the therapeutic mechanisms for hIVIG [81]. While Aβ (oligomers) delivers the kiss of death by 1,000 tiny blows to neurons [82], hIVIG reserves the potential to deliver relief by plethora of mechanisms [83] such as influencing the activation state of microglia, suppressing pro-inflammatory gene expression, immunomodulation, regulating cytokine networks, and fostering neurogenesis in a manner independent of constituent anti-Aβ antibodies. Therefore, enriching hIVIG preparations with anti-Aβ antibodies may not be necessary to unmask the beneficial effects of hIVIG. In view of the limited supply of hIVIG [84] it will be of enormous benefit to further explore the anti-inflammatory, immunomodulatory, and neurogenic mechanisms of hIVIG in the context of Alzheimer’s pathology [85].

## **Conclusions**

We found no *in vivo* support of Aβ clearance from the brains of hIVIG treated transgenic mice either due to disintegration or phagocytosis. We also found no evidence for existence of peripheral sink as mechanism for Aβ clearance in hIVIG treated mice. However, we succeeded to segregate microglia population based on their morphology and Iba-1/CD45 markers, and observed hIVIG treatment effects in these specific subpopulations of microglia. Treatment with hIVIG suppressed TNF-α gene expression and fostered neurogenesis in the dentate gyrus. Our data show limited capacity of hIVIG to alleviate Aβ pathology but provides experimental evidence for immunomodulatory effects.

## **Abbreviations**

Aβ, Amyloid beta; AD, Alzheimer’s disease; Anti-hIgG, Anti-human IgG antibody; hIVIG, Human intravenous immunoglobulin; IFN-α, Interferon alpha; IL-1β, Interleukin 1β; IL-6, Interleukin 6; Nabs-Aβ, Natural Aβ-antibodies; TNF-α, Tumor necrosis factor-α.

## **Competing interests**

HT and JK received funding from Baxter innovations GmbH for carrying out the research project. KO is an employee at Baxter Innovations, Austria. All the experiments and data analysis were done at University of Eastern Finland. Anti-human IgG ELISAs were performed at Baxter Innovations, Austria. The authors have no additional conflicting financial or competing interests.

## **Authors’ contributions**

LP performed mice dosing, immunohistochemistry, confocal microscopy, digital image analysis, data analysis and interpretation, and drafted the manuscript. YP and TM performed RT-PCR and data analysis. KO performed anti-human IgG assay and data analysis. JK and HT participated in study design and coordination, performed data analysis and interpretation, and helped editing the manuscript. All authors read and approved the final manuscript.
